# Gene Therapy for Cystic Fibrosis Lung Disease: Overcoming the Barriers to Translation to the Clinic

**DOI:** 10.3389/fphar.2018.01381

**Published:** 2018-11-27

**Authors:** Martin Donnelley, David W. Parsons

**Affiliations:** ^1^Robinson Research Institute, University of Adelaide, Adelaide, SA, Australia; ^2^Adelaide Medical School, University of Adelaide, Adelaide, SA, Australia; ^3^Respiratory and Sleep Medicine, Women’s and Children’s Hospital, North Adelaide, SA, Australia

**Keywords:** Cystic fibrosis, rat, mouse, airway, lung, genetic therapies, gene-addition, lobe-targeted delivery

## Abstract

Cystic fibrosis (CF) is a progressive, chronic and debilitating genetic disease caused by mutations in the CF Transmembrane-Conductance Regulator (*CFTR*) gene. Unrelenting airway disease begins in infancy and produces a steady deterioration in quality of life, ultimately leading to premature death. While life expectancy has improved, current treatments for CF are neither preventive nor curative. Since the discovery of *CFTR* the vision of correcting the underlying genetic defect – not just treating the symptoms – has been developed to where it is poised to become a transformative technology. Addition of a properly functioning *CFTR* gene into defective airway cells is the only biologically rational way to prevent or treat CF airway disease for *all CFTR* mutation classes. While new gene editing approaches hold exciting promise, airway gene-addition therapy remains the most encouraging therapeutic approach for CF. However, early work has not yet progressed to large-scale clinical trials. For clinical trials to begin in earnest the field must demonstrate that gene therapies are safe in CF lungs; can provide clear health benefits and alter the course of lung disease; can be repeatedly dosed to boost effect; and can be scaled effectively from small animal models into human-sized lungs. Demonstrating the durability of these effects demands relevant CF animal models and accurate and reliable techniques to measure benefit. In this review, illustrated with data from our own studies, we outline recent technological developments and discuss these key questions that we believe must be answered to progress CF airway gene-addition therapies to clinical trials.

## Introduction

Cystic fibrosis (CF) is an insidious disease that slowly smothers the health and potential of too many young lives. It is the most common fatal genetic disease in the developed world; 1 in 25–30 people with Caucasian ancestry carry a single defective copy of the *CFTR* gene (Cystic Fibrosis Foundation, 2018) and have no symptoms; 1 in ∼3000 babies are born with mutations on both alleles, resulting in the disease. CF is a multisystem disease that affects many organs, producing a life expectancy of approximately 40 years (Australian Cystic Fibrosis Data Registry, 2016). Premature death usually results from lung disease, after a lifetime struggling to deal with progressive respiratory failure.

New mutation-specific modulator and corrector pharmaceuticals such as Kalydeco (ivacaftor), Orkambi (lumacaftor/ ivacaftor), and Symdeko (tezacaftor/ ivacaftor) have given substantial benefit to some people with CF. Assuming a patient has the right *CFTR* mutation class, these personalized medicines can increase the presence and/or function of the CFTR protein in the cell, improve lung function, and slow lung disease progression. However, they have been neither preventive nor curative since they do not address the *CFTR* gene defect itself, and some have limited effectiveness. Importantly, the cost of these once-daily medications is prohibitive and likely to be unsustainable, with the health systems in several countries declining to recommend Orkambi for governmental financial support due to poor cost-benefit.

Since CF is a recessive genetic disorder, addition of a single copy of the properly functioning *CFTR* gene into affected CF airway cells is recognized as the only rational and feasible way to prevent or treat CF airway disease (Griesenbach et al., 2016). Gene therapy would provide proper cellular function, regardless of the person’s *CFTR* mutation class. The vision for a child born with CF is that treatment with a proven *CFTR* gene-addition therapy at birth would prevent that child ever developing CF lung disease. For those already living with CF, the same therapy would halt progression of their lung disease.

Gene therapies for a range of inherited diseases have now reached the clinic in China (Kim et al., 2008), Europe (Touchot and Flume, 2017), and most recently in the United States where Luxturna, a gene-addition treatment for retinal dystrophy (Russell et al., 2017) was approved by the FDA in Dec 2017 as a prescription medicine (Morrison, 2018). To 2017, there have been almost 2600 gene therapy trials, including 36 for CF (Ginn et al., 2018). Gene therapy is now a therapeutic reality for some genetic diseases, but the challenge for the CF field is to convert the extensive preclinical developments into an effective and safe treatment option for people with CF.

## Developments in Airway Gene Transfer Techniques

Although gene therapy has the potential to be efficacious for CF, a range of challenges have been identified. Here we describe those challenges and their solutions.

### Vector Designs Affect Efficacy

A 12-month, monthly repeat-dose Phase II *CFTR* non-viral (liposome) gene-transfer clinical trial showed significant, albeit modest and transient, lung function benefits in CF patients (Alton et al., 2015). That study confirmed that a *CFTR* gene therapy can correct human CF lung disease, is likely to be safe, have low immunogenicity, and be amenable to repeated dosing. However, due to the poor efficiency and transient response, that group has since concentrated their development efforts on a more efficient lentiviral (LV) gene vector (Alton et al., 2017). Recently, the delivery of *CFTR* mRNA using nanoparticles has been shown to improve chloride channel function in CF mice for 2 weeks, with a superior response compared to liposomal delivery (Robinson et al., 2018). However, the short duration of action may be a barrier to adoption.

Initial CF gene-addition research used adeno- (Ad) and adeno-associated viruses (AAV) as the gene vectors, but these failed the clinical transition process for CF lung disease due to significant side-effects, and/or lack of efficacy and duration (Moss et al., 2007). AAV also has a DNA packaging capacity limit that requires truncation of the CFTR gene, and AAV vector genomes remain largely episomal (not integrated), meaning that gene transfer to terminally differentiated airway cells is transient and rapidly lost with airway cell turnover (Karda et al., 2016). Despite these challenges [well described by Guggino and Cebotaru (2017)] AAV vector development is continuing, including the assessment of alternative serotypes (Steines et al., 2016; Guggino et al., 2017; Duncan et al., 2018). Interestingly, an integrating *piggyBac*/Ad *CFTR* vector was recently shown to phenotypically correct CF pig airways, with the possibility that it could produce extended gene expression (Cooney et al., 2018).

Lentiviral vectors are now one of the lead CF airway gene-addition vectors for therapeutic development because they transduce dividing and non-dividing cells, can be pseudotyped to target specific cell types by altering surface receptor recognition elements, integrate into the host cell chromosome providing lasting benefit, and generate little immune response. LV vectors have been effective in a range of preclinical studies (Copreni et al., 2004; Cmielewski et al., 2014a; Cooney et al., 2016; Alton et al., 2017). Supported by the clinical successes in effectiveness and safety within *ex vivo* CAR-T cell therapies in cancer treatment (Milone and O’Doherty, 2018), we believe that LV vectors are currently the *CFTR* delivery vehicle of choice. The remainder of this article focuses specifically on the barriers of translating LV gene vectors for CF to the clinic.

### Physical and Biological Barriers of the Airway Surface Restrict Gene Delivery

The airway epithelium has evolved to protect cells against foreign invaders such as viruses and bacteria. The normal physical host defenses at the airway surface play a leading role in limiting the efficiency of any type of gene transfer into the airway epithelium (Kim et al., 2016). Mucus that covers the epithelial surface traps vector particles, which are then removed from the airway by mucociliary clearance (MCC) efficiently preventing vector particles from reaching vector-relevant receptors on the cell membrane surface (Castellani and Conese, 2010). This situation is exacerbated in CF due to the increased volume and viscosity of the mucus (Duncan et al., 2016). Finally, for vectors designed to target the airway stem cells, known to be a subpopulation of airway basal cells at the deep-lying epithelial basement membrane (Rock et al., 2009), access past the epithelial tight junctions is also needed. These tight junctions separate the luminal surface from the deeper layers, acting as a normal defense against pathogenic particles.

Gene vectors must be able to penetrate the CF mucus barrier and reach the underlying target epithelium and target cells. One recognized approach that enables gene vector particles to reach the relevant airway-surface cell vesicular stomatitis virus (VSV-G) receptors and the basal stem cells, is conditioning the airway epithelium with the compound lysophosphatidylcholine (LPC) prior to gene vector delivery. LPC is a normal component of lung surfactant, and its primary role is to transiently permeabilize airway tight-junctions, and it may also help to solubilize surface mucus and enable greater vector access to airway cells. *In vivo* acute and long-term reporter gene studies have shown the effectiveness of LPC conditioning for enhancing gene expression in mice (Limberis et al., 2002; Stocker et al., 2009; Cmielewski et al., 2014a, 2017). In addition, successful gene expression using the LacZ reporter gene has been achieved in short-term (7 day) studies, transducing the conducting airways in sheep (Liu et al., 2010) (a large lung model), marmosets (Farrow et al., 2013) (a primate model), ferrets (Cmielewski et al., 2014b), and rats (McIntyre et al., 2018).

Reporter gene expression is predominantly in ciliated cells, but the LV vector also reaches the alveolar space where type I and II cells and macrophages are transduced. LPC airway conditioning has enabled transduction of endogenous adult airway stem cells (Farrow et al., 2018), confirming the mechanistic basis for stem-cell-based durable *in vivo* gene expression. These studies showed the potential for life-long replenishment of therapeutic benefit via the emergence of cell lineages that were corrected *in situ* by the purposely transduced airway stem cells. LV vectors can also robustly transduce human airway basal cells *in vitro* (Farrow et al., 2018), and human air liquid interface (ALI) cultures, supporting applicability to human airways.

While much understanding can be gleaned from reporter gene studies, the gene transfer methods must also be demonstrated to effectively alter a CF airway, by delivering the *CFTR* gene. Previous studies have shown that LV vectors can partially correct CFTR function in CF mouse nasal airways (the only site of measurable CF airway functional pathophysiology in mice) (Limberis et al., 2002; Stocker et al., 2009; Cmielewski et al., 2014a). Improvement was significant and extended for at least 12 months after the single *CFTR* vector dose, validating the strength and persistence of benefit possible with a LV vector.

The efficacy of LPC enhancement is also not limited to LV vectors. Helper adenoviral vector studies in pigs have showed strong and extensively distributed reporter gene transfer, with no systemic toxicity or infection reported from use of the combined LPC-hAdv vector aerosol (Cao et al., 2013). However, the key question from these studies remains: can a *CFTR* gene-addition process improve the course of disease in a CF lung?

### Suitability of CF Animal Models for CFTR Gene Therapy Research

A range of CF animal models have been developed, with the mouse, pig, ferret and rat the most well characterized (Lavelle et al., 2016; McCarron et al., 2018). Small animal models are ideal for developing gene-addition techniques for CF for cost and handling reasons, and the gene vector volume required to treat the lungs is modest facilitating large well-powered studies. Although many CF mouse models have been developed, the *CFTR* Null mouse does not exhibit lung disease, and the β-ENaC mouse is not suitable for gene-addition therapy development because its lungs contain functional CFTR. The pig (Meyerholz, 2016) and ferret (Sun et al., 2014) models recapitulate human lung disease but exhibit severe gut disease (Meyerholz et al., 2010; Sun et al., 2010) that necessitates intensive husbandry requirements with associated high costs. Lung gene vector dosing requirements prohibit extensive testing in the pig.

Our group recently used CRISPR/Cas9 gene-editing to create colonies of Phe508del (Class II) and 512X CF (Class I) rats (McIntyre et al., 2017). Hallmark CF pathophysiology is consistent with the United States CF rat (Tuggle et al., 2014; Birket et al., 2018), with frequent severe gut obstruction and increased gut motility, as well as poorly developed vas deferens, seminal vesicles & epididymis. As in humans, the phenotype of these two mutations is different. Both have higher mortality than wild-type, with the 512X rats having higher pre- and post-weaning mortality than the Phe508del. Both have a lower average body weight compared to wild-type, although this effect appears to be more pronounced in male animals.

Nasal-airway potential difference (PD) measurements have confirmed altered CFTR airway function in both models. The 512X rats have no response, or a weak response to a chloride-free environment that is consistent with classically defective CFTR function. The Phe508del animals are also significantly different to heterozygote/normal rats, but with an intermediate response consistent with the presence of residual CFTR function seen in the human Phe508del mutation. Since our treatment focus is CF *lung* disease, we are investigating methods of performing PD measurements in the trachea and/or deeper airways, as is possible in humans (Davies et al., 2005), as a method for tracking functional CFTR changes in the CF rat lung.

### Improvements in Airway and Lung Function Must Be Measured

Measuring the effects of airway gene therapies on lung and airway health has been a major challenge for all CF research groups, with CFTR channel function typically assessed using transepithelial potential difference measurements, along with Ussing chamber and halide assays. However, these techniques all have limitations, particularly for *in vivo* use in animal models. Fortunately, Phase Contrast X-ray Imaging (PCXI) based approaches have recently been demonstrated to be able to directly measure airway and lung health *in vivo*.

Phase Contrast X-ray Imaging utilizes X-ray refraction to achieve high spatial and temporal resolution as well as excellent airway and lung soft tissue contrast, and has the potential to dramatically reduce radiation doses (Kitchen et al., 2017). Using PCXI it is now possible to measure changes in airway surface liquid (ASL) depth following treatment (Siu et al., 2008; Morgan et al., 2013, 2016), as well as the clearance of micron-sized marker particles on the airway surface by mucociliary clearance (MCC) (Donnelley et al., 2012a,b, 2014, 2017; Gradl et al., 2018). ASL depth and MCC are the two key airway physiological parameters that must show rapid beneficial changes if a CF airway gene therapy is to be considered effective.

Phase Contrast X-ray Imaging -based analyses have now also been developed to provide quantitative measurement of lung function, taking the focus away from the requirement to use qualitative assessment of structure (i.e., via CT imaging) to *infer* function. Structural changes that occur with CF lung disease alter the flow of air in the lung and the regional patterns of lung motion, whether by obstruction that increases airway resistance, or by changes to the lung parenchyma that alter the mechanical properties of the tissue. It follows that abnormal motion of lung regions during respiration is an accurate indicator of disease (Fouras et al., 2012). Lung motion can be tracked by combining velocimetry techniques with the enhanced airway contrast offered by PCXI. This technique can perform pinpoint spirometry – a method referred to as 4DxV – and was developed and validated in β-ENaC mice at the SPring-8 synchrotron (Stahr et al., 2016). It allows local ventilation to be quantitatively measured at every point in the lung, enabling *local* treatment effects to be assessed. The 4DxV imaging methods can now be used in rat lungs at the Imaging and Medical Beamline at the Australian Synchrotron (Murrie et al., 2015), with commercially available turnkey systems now available (4Dx, Melbourne, VIC, Australia).

With long-lived and easily maintained CF rats now available, and the ability to measure changes in airway surface and lung health in only those lung regions that are treated, it is now possible to quantify the short- and long-term effects of contained, local-region dosing of a gene therapy to the lungs of a living CF animal model, without the need for post-mortem lung analyses.

### Scalable Vector Production and Precision Delivery Capabilities Are Essential

Translating this gene-addition technology to humans requires larger gene vector volumes, but there are a range of challenges associated with upscaling LV vector production (McCarron et al., 2016). Common methods currently employ adherent cell cultures using cell-factories (Rout-Pitt et al., 2018), however, packed-bed bioreactor approaches can now also yield unconcentrated titres of 10^5^–10^6^ TU/ml. Once concentrated and purified for *in vivo* use titres in our laboratory are 10^8^–10^9^ TU/ml. Commercially available, suspension-based production methods such as the LV-MAX^TM^ LV Production System (Gibco) have the potential for scalable production in stirred-tank bioreactors and are animal component-free, important characteristics for establishing LV manufacturing methods for future clinical use (McCarron et al., 2016). In our laboratory unconcentrated LV-MAX^TM^ titres are already greater than 10^6^ TU/ml and are easily scalable to human-sized lung doses by using larger bioreactor vessels. However, until stable packaging cell lines can be used, a major barrier to transient transfection systems remains the production of large quantities of plasmid DNA.

To translate successful preclinical developments into humans it is important to determine whether viral gene therapy techniques developed in small animal models are readily translatable to a human-sized lung. Although intended to be beneficial, these LV gene-addition therapies are deliberately designed to induce permanent genetic alterations. It is the authors’ opinion that the first LV CF gene vector trials in humans will be accompanied by a heightened level of caution compared to new transient-effect daily pharmaceuticals. We propose that the first lung gene-addition trials with viral vectors in humans must be performed bronchoscopically, to enable precise gene delivery and to examine the gene vector effects in a specific limited region of the lung. This method offers an “exit strategy” – by wedge or lobe excision – should a treatment unexpectedly produce unresolvable or unacceptable serious adverse events. However, until recently this strategy could not be tested in small animal models because bronchoscopic delivery was not possible.

The ability to accurately and precisely deliver fluid doses to a small region of the lung has been almost impossible in small animals such as rats and mice due to the small size of their airways. However, the first reliable bronchoscopic dosing technique that can target pre-selected regions of the rat lung was recently reported (McIntyre et al., 2018), and is based on a 1.1 mm diameter rigid sialendoscope normally used for human salivary duct procedures. This miniature bronchoscope system has light, video vision, two access channels, and can be used to dose fluid into at least the fourth-generation branches of the rat airway in ∼200 gram rats. That study showed that rats lungs are amenable to reporter gene delivery and expression at a similar level and with similar cell type distributions across nasal and lung regions as found in mouse studies. *LacZ* expression was present in ciliated and goblet / secretory cells, the two most relevant cell populations on the conducting-airway surface epithelium. However, unlike nasal or tracheal delivery, bronchoscopic delivery limited transduction to only the treated lung lobe/region.

Together, bronchoscopic dosing and bioreactor production methods allow the developments in small animal models to be easily scaled to the levels required for translational to human clinical trials.

## The Main Challenges for the Field

An effective gene-addition therapy for CF lung disease requires accurate compound delivery to the target location, high levels of transduction, and effective CFTR protein expression in the cells relevant to CF disease. Reliable and relevant measurements of the benefit to the treated region, as well as the whole lung, are essential. However, several challenges remain before genetic therapies for CF lung disease can be translated to the clinic.

### Gene Therapy Efficacy Should Be Validated in a CF Lung

The ability of a viral gene-addition vector to adequately modify CF lung disease health and progression has never been demonstrated, but it is the authors’ opinion that this is essential prior to human clinical trials. Certainly, the effectiveness of *CFTR* gene-addition *per se* has been validated clinically by the UK CF Gene Therapy Consortium (Alton et al., 2015), and that group has undertaken extensive preparation for clinical trials of lung *CFTR* gene-addition using a SIV LV vector. Although the consortium has not yet reported *in vivo* CFTR functional improvements in a CF animal model, they have approval for human trials (Alton et al., 2017). Similarly, the Iowa United States CF gene therapy research group have used uninfected neonatal CF pigs to examine LV-*CFTR* gene transfer efficacy, but the absence of suitable outcome measures (as described in the previous section) meant that the CF pigs were necessarily humanely killed 2 weeks later for lung-tissue analysis (Cooney et al., 2016). The ability to perform localized delivery of LV-*CFTR* into a diseased CF rat lung, combined with non-invasive PCXI based assessments is expected to enable the first-ever *in vivo* long-duration testing of LV gene addition therapy for CF lung disease. Future long-term studies should also assess survival differences produced by *CFTR* gene-addition, to estimate the likely human therapeutic benefit.

### The Safety Profile of LPC and LV Must Be Demonstrated in a CF Lung

The power of gene therapy was illustrated following clinical trials using first generation γ-retroviral vectors, which successfully treated genetic disorders such as SCID-X1. However, they resulted in serious adverse events such as acute lymphoblastic leukemia due to viral promoters that upregulated proto-oncogenes lying close to the integration site (Hacein-Bey-Abina et al., 2008; Howe et al., 2008). LV vectors were subsequently redesigned to improve their safety profile. Those modifications included reducing the amount of native HIV genome in the vector and modifying the viral long terminal repeat (LTR) to remove endogenous viral promoter sequences to render the vectors replication deficient (Yu et al., 1986; Dull et al., 1998). Finally, the viral components required for replication were separated onto separate plasmids and delivered *in trans*. Nonetheless, comprehensive integration site analyses remain essential for vectors destined for clinical trials.

The safety of airway conditioning compounds must also be established. LPC action is transient, dose-controllable, and well-tolerated in *in vivo* animal studies. Despite demonstrating that LPC enables effective gene-addition and is well-tolerated in multiple animal species, it is not known whether the desired transient tight-junction permeabilization in a CF airway, where infective bacteria are normally present, may bring unacceptable risks. Potentially allowing those pathogens access to normally protected sub-surface regions, raises the possibility of additional local or even systemic infections to occur. The theoretical dangers of altering airway barrier function, even transiently, in the infective milieu of a CF lung are obvious. However, LPC has a convincing preclinical history of safe use, so the potential of airway conditioning and gene-addition therapy in humans with CF must not simply be dismissed as too risky in the absence of any evidence. The rule *primum nil nocere*, first do no harm (Smith, 2005), which drives much of the bioethics of medicine, it is not an absolute. For example, acceptance of the harm, pain, and risk from myeloablative conditioning is accepted as an essential conditioning procedure for bone marrow transplantation. These important questions related to the acceptability of airway / lung conditioning are yet to be answered.

### Methods to Increase Gene Expression Levels Must Be Developed

The ability to achieve reporter or *CFTR* gene transfer using LV vectors has previously been established, with *in situ* transduction of resident airway basal cells that replenish the airway with transgene-expressing daughter cells the likely basis for this long-term expression. However, in most mice gene expression eventually wanes, and in some reduces to zero after only 6-months (Cmielewski et al., 2014a). This data, and the inherent uncertainties of translating *in vivo* mouse data to a human disease, suggests that it may be beneficial or necessary to produce higher levels of gene expression through initial multi-dosing, or by repeat-dosing if gene expression wanes. However, a host immune response directed against the gene transfer agent may block initial gene expression and/or prevent expression arising from repeated doses. Few studies have examined strategies for effectively re-administering LV vectors to the airways.

Feline immunodeficiency virus (FIV) and simian immunodeficiency virus (SIV) LV vectors have been successfully re-administered to the airways without loss of effectiveness, suggesting LV vectors may evade adaptive immune responses (Sinn et al., 2008; Griesenbach et al., 2012). These vectors were pseudotyped with the GP64 and F/HN envelopes, respectively, which both target receptors located on the apical surface of the airway, so they are unlikely to be able to reach or transduce the basal stem cells located on the basolateral airway surface. The SIV study showed repeat dosing was feasible but did not increase transgene expression. These studies also highlighted that expression levels are highly dependent on the dose timing and the choice of transgene delivered at each dose. The knowledge that patients will continue to benefit from additional doses will revolutionize CF airway gene therapy and pathways to clinical trials.

### Dosing Methods Need to Be Optimized in Human-Sized Lungs

The ability to dose human sized lungs must also be established early. Human lungs are substantially larger than in small animal models. They are also differently developed and branched, and cell type distributions and proportions along the conducting airways where CF pathophysiology begins are different. Confirming the feasibility and effectiveness of LV gene-addition techniques in human-sized lungs, such as in pigs or sheep, along with the necessary vector production requirements, is of vital importance.

## Conclusion

While expensive daily drug-based options for treating the downstream effects of the *CFTR* gene defect are emerging, gene-addition therapies are the only approach with the immediate potential to prevent or halt CF lung disease. Although treatment for pancreatic insufficiency, CF related diabetes and other CF associated pathologies will still be required, by dealing with the fundamental gene defect at its source within airway cells, airway gene therapy can be expected to transform the treatment options for CF lung disease.

A range of proven LV gene vectors and adequate novel vector production techniques have been developed. LPC and LV gene-addition techniques have already been validated in multiple animal models. The production of new animal models such as the CF rat will enable significant advances in CF translational capability, with bronchoscopic delivery techniques able to dose individual lobes of the rat lung. Innovative new non-invasive PCXI-based measurements of airway and lung function can now be used to complement standard measurement techniques.

It is the authors’ opinions that key questions that must be answered before airway gene therapy can be translated to the clinic involve clearly demonstrating long-term efficacy and the safety of LPC and LV delivery in a CF lung, the ability to re-dose to boost *CFTR* gene expression levels, and the ability to translate these techniques into human-sized lungs. Together the new capabilities described here will allow these key questions that will enable the translation of airway gene therapy to humans to be answered.

## Author Contributions

DP and MD contributed to the conception and design of the work and wrote the manuscript.

## Conflict of Interest Statement

DP is a current shareholder of 4Dx Ltd. He has no financial benefit relationships with 4Dx Ltd. The handling Editor declared a past collaboration with one of the authors DP. The remaining author declares that the research was conducted in the absence of any commercial or financial relationships that could be construed as a potential conflict of interest.
